# Mental health literacy of Chinese nurses from public general and psychiatric hospitals: a cross-sectional study

**DOI:** 10.3389/fpsyt.2023.1148380

**Published:** 2023-08-01

**Authors:** Anni Wang, Shoumei Jia, Zhongying Shi, Xiaoming Sun, Yuan Zhu, Miaoli Shen, Dayu Tang, Xizhu Chen

**Affiliations:** ^1^School of Nursing, Fudan University, Shanghai, China; ^2^Shanghai Mental Health Centre, Shanghai, China; ^3^Shanghai Tongji Hospital, Shanghai, China; ^4^Shanghai Chest Hospital, Shanghai, China; ^5^Changning District Mental Health Center, Shanghai, China

**Keywords:** mental health literacy, nurses, health professional, general hospital, psychiatric hospital

## Abstract

**Background:**

Mental health literacy (MHL) is crucial to address issues related to mental illness. Nurses’ MHL is even more important because they are expected to deal with both the physical and psychological consequences of mental disorders.

**Objective:**

This study investigated the level, discrepancy, and characteristics of MHL among Chinese nurses from both public general and psychiatric hospitals; identified influential factors; and explored the relationship between MHL and mental health status.

**Methods:**

Using a stratified cluster sampling method to select participants, a cross-sectional survey was conducted to describe the MHL of 777 nurses from 13 general and 12 psychiatric hospitals using the Chinese version of the Mental Health Literacy Scale, Patient Health Questionnaire-2, Generalized Anxiety Disorder-2, and a demographic questionnaire. A multiple regression analysis was used to determine the factors influencing MHL among the nurses recruited.

**Results:**

The participants’ total score on the Chinese version of the Mental Health Literacy Scale was 93.25 (SD = 10.52). Multiple regression analysis revealed that nurses who worked in psychiatric or higher-level hospitals, with higher professional titles or higher education had higher levels of overall MHL and core MHL, while those working in general hospitals, with shorter work duration, or who were unmarried had higher social acceptance of patients. Nurses’ MHL was closely correlated with their mental health status.

**Conclusion:**

The overall and core MHL of Chinese nurses were at a moderate level, with social acceptance remaining at a relatively low level. There is an urgent need for MHL promotion programs to improve the MHL of clinical nurses. The focus must be given to overall MHL, especially core MHL, for non-psychiatric nurses to enhance their competence in mental health promotion and identification; more emphasis should be placed on the social acceptance of patients with mental illnesses for psychiatric nurses to improve their provision of professional services. Better MHL would be a formula for improving nurses’ own mental health and their mental health service competence.

## 1. Introduction

Mental health conditions are increasing worldwide, have gradually become one of the leading causes of disease over the past 30 years, and have significantly impacted the quality of life of individuals and communities. Mental disorders and behavioral problems have also become more common across China in recent decades, with 5.81 million patients having serious mental illnesses by the end of 2017 (1). The data show that the weighted lifetime prevalence of various mental disorders (excluding dementia) is 16.6% (2). Mental health problems lead to adverse health outcomes, premature death, and economic losses, and have become an important social and public health issue. Measured by years lived with disability, the burden of mental disorders exceeds 1/5th of the global disease burden (GDB), with depression being among the top three diseases in the GDB (3).

One of the most important reasons for the prevalence of mental health problems is that public mental health literacy (MHL) is generally low (4–8). In the literature on health literacy, Jorm et al. (4) first proposed the concept of MHL to broadly refer to knowledge and attitudes regarding mental health that aid in the recognition, management, and prevention of mental health issues. MHL is a multifaceted concept that consists of the following key attributes: The ability to recognize specific disorders, knowing how to seek mental health information, knowledge of risk factors and causes, knowledge of self-treatment, knowledge of available professional help, and attitudes that promote recognition and appropriate help-seeking (4, 9). O’Connor et al. (9) further classified the attributes of MHL into three substructures: Recognition, knowledge, and attitude. Similarly, Jiang et al. (10) defined MHL as the knowledge, attitudes, and behaviors that aid in mental health promotion and coping with mental illness for the self and others. A comprehensive definition of the multiple components of MHL would provide a basis for research and practice to promote mental health in society.

MHL is considered a prerequisite for early recognition and intervention in mental disorders with the potential to improve both individual and community health. Accurate recognition of a mental illness and its perceived severity increases the need for care and treatment by psychiatrists and mental health professionals, while poor MHL may prevent or deter individuals from using appropriate treatment options (11–13). Improved knowledge about mental health and mental disorders; better awareness of how to seek help and treatment; and reduced stigmatization of mental illness at the individual, community, and institutional levels may promote early identification of mental disorders (14), lead to greater intention to seek help (6, 15), reduce suicide risk (16), increase the use of health services and positive attitudes toward treatment (6, 17), and improve mental health outcomes and quality of life (14, 18–21). Low levels of MHL are associated with negative attitudes toward psychological treatment and are always coupled with ineffective or non-professional help-seeking preferences (17, 22). Individuals with conditions such as anxiety and mood disorders often do not receive any treatment in their initial years, and many never seek or receive appropriate treatment (13, 23, 24).

While MHL is crucial in addressing mental illness issues, studies have shown low levels of MHL in a diverse range of populations, including a lack of knowledge about prevention, development, and feasible treatments for mental illness and how to seek, help, or assist individuals with mental health problems (6, 15, 25). According to a national mental health assessment conducted by the Psychological Institute of the Chinese Academy of Science, the baseline MHL in China was 12% (19). A recent study in China found that national MHL is at a medium-low level in citizens aged 18 and above, with mental health maintenance and promotion literacy being higher than mental illness coping literacy, and self-help literacy being higher than helping people literacy (26). In an MHL survey in three cities in China, it was found that the awareness rate of mental health knowledge was 49.8%, and the understanding of mental diseases and their symptoms was inadequate (27). Other studies have also shown that the public has a low identification rate for mental illness, a lack of initiative in seeking professional treatment or inappropriately seeking help, and serious stigma (28–30). Consequently, given the relatively high prevalence of mental disorders, the low level of MHL in China has imposed a huge challenge and burden on society.

The level of MHL among healthcare professionals (HCPs) is even more important because it shapes the therapeutic relationships in which they work in partnership with patients. As people who are in close contact with patients and their families, the MHL of nurses is crucial not only in providing prompt recognition and appropriate referrals but also in influencing their attitudes toward people with mental illness (13) and promoting the mental health of the population (31, 32). Given their academic background and professional training, nurses are expected to deal with both the physical and psychological consequences of mental disorders.

However, despite the general perception that HCPs are more equipped and sympathetic toward patients with mental illnesses, knowledge about mental health and illness is still lacking (33). Simultaneously, HCPs who suffer from mental problems do not seek help or speak to colleagues about their issues because of the stigmatizing culture (33). Several studies have revealed that HCPs have limited knowledge of mental health issues, and many practitioners exhibit a common notion of feeling incompetent and discouraged about the management and recovery of mentally ill individuals (32). A recent survey in China showed that the MHL of non-psychiatric nurses is inadequate, with correct recognition rates of schizophrenia, depression, and generalized anxiety being 38.9, 56.2, and 17.5%, respectively (13). To date, the level of MHL among psychiatric nurses and the discrepancy in MHL between psychiatric and non-psychiatric nurses remain unclear (12, 13). Furthermore, the key attributes of MHL among these two groups also need to be addressed (4, 9, 10), which is essential for future targeted promotion strategies.

The present study is the first trial aimed at addressing the level and characteristics of MHL among Chinese nurses from public general and psychiatric hospitals, identifying influential factors, and exploring the relationship between MHL and mental health status. The results will aid in action plans for improving the MHL and mental health levels of nurses and health professionals.

## 2. Methods

### 2.1. Design

This was a cross-sectional study on MHL among nurses from public general and psychiatric hospitals in Shanghai, China.

### 2.2. Participants

Using stratified cluster sampling, 12 of the 21 psychiatric hospitals (including 2 tertiary and 10 secondary public hospitals) and 13 of the 75 general hospitals (including 3 tertiary and 10 secondary public hospitals) in Shanghai were selected. Two to three wards from each hospital were randomly selected. This sampling plan was designed by combining a pilot investigation and formula calculation. First, based on simple random sampling size formula of $n_{1}=\frac{{\left(Z_{\frac{\alpha }{2}}\sigma \right)}^{2}}{\delta^{2}}$, we set *σ* = 9.81, *δ* = 2 based on pilot data of 30 psychiatric nurses. The *n*_1_ value was calculated to be 92. Then, based on the cluster sampling sample size formula *n*_2_ = deff×*n*_1_, we set the deff value to the regular high value of 3. The *n*_2_ was calculated to be 276. Adding an estimated dropout rate of 20–25%, 345–368 psychiatric nurses should be reached. Each ward had approximately 15 nurses and we planned to select two wards in each hospital. Thus, 24 wards in 12 psychiatric hospitals were needed. Based on the distribution of psychiatric hospitals at different levels in Shanghai, we selected two tertiary hospitals and 10 secondary hospitals. For nonpsychiatric nurses, the procedure was the same as described above. Based on *σ* = 10.40, *δ* = 2 of pilot non-psychiatric nurses, a total of 26 units from 13 hospitals were needed. All nurses in the selected wards who met the inclusion criteria were invited to participate in this study with the help of a head nurse during ward meetings. The inclusion criteria were as follows: (1) Registered nurses with working experiences of ≥1 year, (2) aged 18–60 years old, and (3) consenting to participate in the study. The exclusion criteria were as follows: (1) Nurses with severe mental illnesses, such as schizophrenia and other psychotic disturbances, and (2) trainee nurses or those with nursing student status.

### 2.3. Measures

The instruments included the validated Chinese version of the Mental Health Literacy Scale (MHLS-C), Patient Health Questionnaire-2 (PHQ-2), Generalized Anxiety Disorder-2 (GAD-2), and a demographic questionnaire.

#### 2.3.1. MHLS-C

This scale was developed by Matt O’Connor in 2015, is based theoretically on the concept of MHL, and contains 35 items (34). Before this study, with the consent of the original author of the scale, the MHLS was translated into a Chinese version (MHLS-C) by the research team according to the Brislin translation model, and the construct validity of the MHLS-C was tested to have 29 items with four factors: “Knowledge of mental disorder” (knowledge, 13 items), “ability to seek information and help” (ability, four items), “recognition of mental disorder” (recognition, five items) and “acceptance of patients with mental illness” (acceptance, seven items). Factors one to three were summarized into the MHLS-Core (core literacy subscale), and factor four was taken as the MHLS-SA (social acceptance subscale), which could be used independently (35). The Cronbach’s alpha coefficients of MHLS-C, MHLS-Core, and MHLS-SA were 0.85, 0.89, and 0.93 respectively; the test-retest reliability of MHLS-C was also good, with intraclass correlation coefficients (ICCs) of 0.80 for the whole scale and 0.79 and 0.94 for two subscales (35). The total scores range from 29 to 132, with higher scores indicating better MHL.

#### 2.3.2. PHQ-2

The PHQ-2 is composed of the first two questions of the PHQ-9, which was specifically developed as a screening instrument for depressive symptoms experienced during the preceding 2 weeks. Questions were measured using a 4-point Likert scale (0 = not at all, 1 = several days, 2 = more than half of the days, and 3 = nearly every day). The total score of the PHQ-2 ranges from 0 to 6, with higher scores indicating greater severity of depressive symptoms. A cutoff of 3 for the PHQ-2 has demonstrated satisfactory sensitivity (81%) and specificity (96%) for screening depression (36). In this study, the Cronbach’s alpha coefficient for the PHQ-2 was 0.83.

#### 2.3.3. GAD-2

The GAD-2 is composed of the first two questions from the GAD-7, which was specifically developed as a screening instrument for anxiety symptoms experienced during the preceding 2 weeks. Questions were measured using a 4-point Likert scale (0 = not at all, 1 = several days, 2 = more than half of the days, and 3 = nearly every day). The total GAD-2 score ranged from 0 to 6, with higher scores indicating more anxiety symptoms. A cutoff of 3 for the GAD-2 is acceptable for identifying anxiety disorders with 76% sensitivity and 81% specificity (37). In this study, the Cronbach’s alpha coefficient for the GAD-2 was 0.87.

The demographic questionnaire included the following items: Sex, age, education, marital status, work duration, professional title and position, sleep quality, and physical condition.

### 2.4. Procedures

A cross-sectional online survey was conducted with the approval of the nursing directors of the selected hospitals and assistant head nurses in each ward. The investigator distributed the e-invitation and questionnaire link via the widely used smartphone-based investigation tool Wenjuanxing, which was sent to the nurses recruited by a head nurse. All respondents provided informed consent electronically and were made aware of the confidentiality and voluntary nature of the study before participation. The average time to complete the questionnaires was 5–10 min. No individually identifiable information was collected or stored during the process. Data were collected in April 2021.

### 2.5. Statistics

Data were analyzed using SPSS version 22.0. Data were exported from the Questionnaire Star software and initially examined for distribution normality and outliers. Questionnaires with a completion time of less than 2 min were excluded. The frequency, mean, and standard deviation (SDs) were calculated for the demographic data and scores of the MHLS-C, PHQ-2, and GAD-2. Independent sample *t*-tests, chi-square tests, and Pearson correlations were used to examine the relationships between nurses’ characteristics, mental health, and MHL and to compare the MHL of nurses between general hospitals and psychiatric hospitals. A multiple linear regression model was used to explore factors influencing nurses’ MHL in both general and psychiatric hospitals, which based on dependent variables’ not normal distribution in the Kolmogorov Smirnov test, but displaying approximately normal distribution in the histogram. Fewer outliers were also included for these were nurses’ actual reactions and experiences. For all statistical analyses, the alpha value was set at 0.05.

## 3. Results

### 3.1. Participants’ characteristics

In total, 777 nurses were recruited; 476 were from general hospitals and 301 were from psychiatric hospitals; 209 were from tertiary and 568 were from secondary hospitals. The mean age of the participants was 35.19 years (SD = 8.74), with nurses in psychiatric hospitals being slightly older and working longer than those in general hospitals. Most nurses were female (751, 96.7%), with higher male nurse ratio in psychiatric hospitals. Two-thirds of the nurses (516, 66.4%) were married and a few more were divorced in psychiatric hospitals. More than half of the nurses had a bachelor’s degree or higher (476, 61.3%), and their work experience ranged from 1 to 37 years. Most nurses (660, 84.9%) were involved in clinical practice, while others were engaged in nursing management. Most nurses (*N* = 573, 73.7%) had junior professional titles. Approximately one-quarter of the nurses (196, 25.2%) reported poor sleep quality, whereas others reported moderate or good quality. More than one-third (299, 38.5%) had at least one chronic health problem such as diabetes or hypertension. There were no differences between nurses in general and psychiatric hospitals in terms of education, professional title and position, chronic problems, or sleep quality (Table 1).

**Table 1:** Demographics of nurses in public general and psychiatric hospitals (*n* = 777).

Items		Total	General hospitals (*n* = 476)	Psychiatric hospitals (*n* = 301)	*T*/χ^2^	*p*
Age		35.19 ± 8.74	34.48 ± 8.45	36.33 ± 9.08	−2.836	0.005
Sex	Male	26 (3.3%)	8 (1.7%)	18 (6.0%)	10.539	0.001
	Female	751 (96.7%)	468 (98.3%)	283 (94.0%)		
Education	Technical secondary school degree	35 (4.5%)	16 (3.4%)	19 (6.3%)	6.156	0.104
	College diploma	266 (34.2%)	155 (32.6%)	111 (36.9%)		
	Bachelor’s degree	462 (59.5%)	296 (62.2%)	166 (55.1%)		
	Graduate degree	14 (1.8%)	9 (1.9%)	5 (1.7%)		
Marital status	Unmarried	240 (30.9%)	159 (33.4%)	81 (26.9%)	9.723	0.008
	Married	516 (66.4%)	310 (65.1%)	206 (68.4%)		
	Divorced	21 (2.7%)	7 (1.5%)	14 (4.7%)		
Hospital level	Tertiary	209 (26.9%)	125 (26.3%)	84 (27.9%)	0.096	0.757
	Secondary	568 (73.1%)	351 (73.7%)	217 (72.1%)		
Work duration	No more than 5 years	176 (22.7%)	116 (24.4%)	60 (19.9%)	18.607	0.000
	6–10 years	159 (20.5%)	97 (20.4%)	62 (20.6%)		
	11–20 years	229 (29.5%)	157 (33.0%)	72 (23.9%)		
	More than 20 years	213 (27.4%)	106 (22.3%)	107 (35.5%)		
Professional title	Junior level nurse	573 (73.7%)	347 (72.9%)	226 (75.1%)	5.499	0.064
	Medium level nurse	184 (23.7%)	121 (25.4%)	63 (20.9%)		
	Senior level nurse	20 (2.6%)	8 (1.7%)	12 (4.0%)		
Professional position	Management nurse	117 (15.1%)	79 (16.6%)	38 (12.6%)	2.275	0.132
	Clinical nurse	660 (84.9%)	397 (83.4%)	263 (87.4%)		
Chronic problems	None	478 (61.5%)	295 (62.0%)	183 (60.8%)	0.474	0.789
	One health problem	226 (29.1%)	139 (29.2%)	87 (28.9%)		
	Two or more health problems	73 (9.4%)	42 (8.8%)	31 (10.3%)		
Sleeping quality	Poor	196 (25.2%)	108 (22.7%)	88 (29.2%)	4.203	0.122
	Moderate	402 (51.7%)	254 (53.4%)	148 (49.2%)		
	Good	179 (23.0%)	114 (23.9%)	65 (21.6%)		

### 3.2. The MHL level and mental health of nurses in general and psychiatric hospitals

The MHLS-C score of all nurses was 93.25 (SD = 10.52), with MHLS-Core and MHLS-SA being 73.57 (SD = 9.19) and 19.68 (SD = 5.33), respectively. Using the ratio of the total score and mean score to illustrate the MHL level, the nurses had a moderate level of MHL (70.6) and MHLS-Core (75.8%), with knowledge being the best (79.1%) and MHL-SA the lowest (56.2%). The MHLS-C and MHLS-Core with the dimensions of knowledge, ability, and recognition of psychiatric nurses were significantly better than those of general nurses, whereas the MHLS-SA was lower than that of general nurses (Table 2). It was also found that general nurses had higher anxiety levels than psychiatric nurses (Table 2).

**Table 2:** The MHL of nurses in public general and psychiatric hospitals (*n* = 777).

Items	Total score	Average score	Scoring rate (%)	General hospitals (*n* = 476)	Psychiatric hospitals (*n* = 301)	*T*	*p*
Knowledge	52	41.11 ± 5.66	79.1	40.35 ± 5.55	42.32 ± 5.62	−4.802	<0.001
Ability	20	14.90 ± 2.96	74.5	14.41 ± 2.85	15.67 ± 2.97	−5.876	<0.001
Recognition	25	17.56 ± 4.04	70.2	16.93 ± 3.97	18.54 ± 3.95	−5.497	<0.001
MHLS-Core	97	73.57 ± 9.19	75.8	71.70 ± 8.37	76.53 ± 9.66	−7.378	<0.001
MHLS-SA	35	19.68 ± 5.33	56.2	20.19 ± 5.43	18.89 ± 5.09	3.336	0.001
MHLS-C	132	93.25 ± 10.52	70.6	91.89 ± 9.79	95.42 ± 11.27	−4.613	<0.001
PHQ-2	6	1.95 ± 1.47	–	2.01 ± 1.47	1.86 ± 1.46	1.382	0.168
GAD-2	6	1.91 ± 1.53	–	2.01 ± 1.50	1.76 ± 1.56	2.254	0.024

### 3.3. Influential factors of the MHL of nurses in general and psychiatric hospitals

Univariate analysis showed that besides the factors of hospital type, nurses with older age, higher education, longer work duration, higher professional title or management position, better sleep quality, working in higher-level hospitals, or being divorced had a higher MHLS-Core. Factors that predicted better MHLS-SA scores among nurses included younger age, higher education, shorter work duration, and being unmarried. Moreover, nurses with higher education, higher hospital level, higher professional titles or management positions, better sleep quality, and divorced status had higher MHLS-C scores (Table 3).

**Table 3:** Comparison of MHL among different groups of nurses (*n* = 777).

Items		MHLS-Core	*p*	MHLS-SA	*p*	MHLS-C	*p*
Age		*r* = 0.087	0.016	*r* = −0.180	<0.001	*r* = −0.015	0.671
Sex	Male	72.81 ± 8.92	0.668	19.35 ± 6.12	0.742	92.15 ± 11.99	0.588
	Female	73.60 ± 9.21		19.70 ± 5.31		93.29 ± 10.48	
Education	Technical secondary school degree	69.31 ± 10.54	<0.001	18.31 ± 4.76	0.043	87.63 ± 11.83	<0.001
	College diploma	72.33 ± 9.07		19.88 ± 5.63		92.22 ± 10.44	
	Bachelor’s degree	74.49 ± 9.02		19.66 ± 5.21		94.15 ± 10.29	
	Graduate degree	77.29 ± 8.25		20.29 ± 5.01		97.57 ± 10.61	
Marital status	Unmarried	73.08 ± 9.31	0.010	21.06 ± 5.01	<0.001	94.13 ± 10.74	0.036
	Married	73.56 ± 9.12		19.11 ± 5.33		92.67 ± 10.44	
	Divorced	79.43 ± 7.93		18.05 ± 6.16		97.48 ± 8.61	
Hospital level	Tertiary	74.69 ± 8.82	0.039	20.06 ± 5.20	0.232	94.75 ± 10.17	0.016
	Secondary	73.16 ± 9.30		19.55 ± 5.38		92.70 ± 10.61	
Work duration	No more than 5 years	72.16 ± 9.18	0.011	21.30 ± 5.22	<0.001	93.47 ± 10.67	0.898
	6–10 years	73.58 ± 8.94		19.73 ± 4.86		93.31 ± 10.20	
	11–20 years	73.15 ± 9.25		19.67 ± 5.48		92.82 ± 10.82	
	More than 20 years	75.17 ± 9.15		18.33 ± 5.27		93.51 ± 10.37	
Professional title	Junior level nurse	72.42 ± 9.01	<0.001	19.82 ± 5.37	0.432	92.24 ± 10.31	<0.001
	Medium level nurse	76.59 ± 8.92		19.36 ± 5.16		95.95 ± 10.53	
	Senior level nurse	78.70 ± 9.36		18.75 ± 5.90		97.45 ± 11.56	
Professional position	Management nurse	76.31 ± 8.89	<0.001	19.32 ± 5.32	0.429	95.63 ± 10.20	0.008
	Clinical nurse	73.08 ± 9.17		19.75 ± 5.34		92.83 ± 10.53	
Chronic problems	None	73.80 ± 9.41	0.505	19.68 ± 5.25	0.133	93.48 ± 10.72	0.184
	One health problem	73.42 ± 9.02		20.04 ± 5.36		93.46 ± 10.57	
	Two or more health problems	72.49 ± 8.28		18.60 ± 5.68		91.10 ± 8.77	
Sleep quality	Poor	72.69 ± 8.81	0.002	19.70 ± 5.27	0.648	92.39 ± 10.28	0.002
	Moderate	73.05 ± 9.26		19.54 ± 5.59		92.60 ± 10.53	
	Good	75.68 ± 9.19		19.99 ± 4.80		95.67 ± 10.47	

Multiple regression analysis was used to determine the factors influencing MHL in nurses of general and psychiatric hospitals, with MHLS-Core, MHLS-SA, and MHLS-C as the dependent variables and the above statistically significant factors during univariate analysis as the independent variables. The dependent variables were approximately normally distributed in the histogram. Nurses who worked in psychiatric (β = 1.958, *p* < 0.001; β = 2.518, *p* < 0.001) or higher-level hospitals (β = −1.998, *p* < 0.05; β = −1.735, *p* < 0.05), with higher professional titles (β = 1.789, *p* < 0.001; β = 1.809, *p* < 0.001) or higher education (β = 2.050, *p* = 0.001; β = 1.884, *p* < 0.001) had higher MHLS-C and MHLS-Core scores, while those working in general hospitals (β = −0.539, *p* < 0.01), with shorter work duration (β = −0.596, *p* < 0.01), or who were unmarried (β = −0.964, *p* < 0.05) had higher MHLS-SA scores (Table 4).

**Table 4:** Multiple linear regression analysis of MHL in nurses (*n* = 777).

	*β*	Std. error	Standardized β	*T*	*p*	95% CI	
						Lower	Upper
**MHLS-Core**							
Hospital type	2.518	0.317	0.267	7.940	<0.001	1.896	3.141
Professional title	1.809	0.346	0.184	5.231	<0.001	1.130	2.488
Education	1.884	0.533	0.125	3.533	<0.001	0.837	2.931
Hospital level	−1.735	0.696	−0.084	−2.493	0.013	−3.102	−0.369
**MHLS-SA**							
Work duration	−0.596	0.207	−0.124	−2.877	0.004	−1.003	−0.189
Hospital type	−0.539	0.193	−0.099	−2.795	0.005	−0.917	−0.160
Marital status	−0.964	0.455	−0.092	−2.118	0.034	−1.858	−0.071
**MHLS-C**							
Hospital type	1.958	0.376	0.181	5.214	<0.001	1.221	2.695
Professional title	1.789	0.424	0.159	4.215	<0.001	0.956	2.622
Education	2.050	0.629	0.118	3.259	0.001	0.815	3.285
Hospital level	−1.998	0.827	−0.084	−2.417	0.016	−3.621	−0.375

For MHLS-Core, *R*^2^ was 0.133 (*F* = 29.679, *p* < 0.001); for MHLS-SA, *R*^2^ was 0.051 (*F* = 13.833, *p* < 0.001); for MHLS-C, *R*^2^ was 0.083 (*F* = 13.913, *p* < 0.001).

### 3.4. Relationships between MHL and the mental health of hospital nurses

Pearson correlation analysis showed that both the PHQ-2 and GAD-2 were significantly negatively correlated with the MHLS-Core, ability, and MHLS-C, with recognition also related to nurses’ PHQ-2. For general nurses, both PHQ-2 and GAD-2 were significantly negatively correlated with the MHLS-Core and its dimensions of ability and recognition. For psychiatric nurses, PHQ-2 was significantly negatively correlated with MHLS-C, MHLS-Core, MHLS-SA, ability, and recognition, and GAD-2 was negatively correlated with the ability and MHLS-SA (Table 5).

**Table 5:** The relationships between MHL and mental health of nurses (*n* = 777).

	Mental health	Knowledge	Ability	Recognition	MHLS-Core	MHLS-SA	MHLS-C
All nurse participants	PHQ-2	0.037	−0.255^**^	−0.201^**^	−0.147^**^	−0.020	−0.138^**^
	GAD-2	0.069	−0.226^**^	−0.106	−0.179^**^	−0.007	−0.099^**^
Nurses in general hospitals (*n* = 476)	PHQ-2	0.063	−0.216^**^	−0.216^**^	−0.135^**^	0.061	−0.081
	GAD-2	0.070	−0.198^**^	−0.207^**^	−0.119^**^	0.046	−0.076
Nurses in psychiatric hospitals (*n* = 301)	PHQ-2	0.022	−0.304^**^	−0.161^**^	−0.147^*^	−0.174^**^	−0.204^**^
	GAD-2	0.106	−0.239^**^	−0.106	−0.055	−0.119^*^	−0.101

*p < 0.05; **p < 0.01.

## 4. Discussion

Nurses are medical staff members who are in direct contact with patients and their families, and have more opportunities to observe them. Therefore, nurses’ MHL is essential to promote the mental health of patients, their families, and communities.

### 4.1. The overall MHL in Chinese nurses

This study measured the main constituents of MHL among Chinese nurses. According to the ratio of the total score to the mean score, the overall MHL (70.6%) and MHL-Core scores were moderate (75.8%), and the social MHL-SA remained at a relatively low level (56.2%), a finding that coincided with that of other studies (13, 32, 33).

In terms of Core MHL, the results showed that nurses’ knowledge of mental illness was relatively good (79.1%), and this knowledge advantage lays the foundation for identifying a range of common mental disorders, such as depression and anxiety. However, this study found that nurses’ “ability to seek help” (74.5%) and “recognize mental illness” (70.2%) were at a moderate level. These results indicate that the negative social stigma toward mental illness among Chinese populations might contribute to a reluctance to associate symptoms with mental illness and label the individual as having a mental disorder (27–30). Instead, there is a greater tendency to attribute socially or culturally appropriate labels (e.g., work problems) to mental symptoms (2). Although early help-seeking for mental illness has been shown to promote timely intervention and improve long-term outcomes (30), the main intrapersonal barriers to mental health help-seeking exist because those with mental illness or symptoms feel ashamed and opt to conceal their condition, preferring to handle their problems by themselves (38, 39). The inadequate ability of nurses to recognize and seek help may lead to a delay in the effective treatment of mental illnesses and an inability to provide help for patients.

Moreover, nurses had a low social acceptance (MHLS-SA) of patients with mental illnesses. This is consistent with other results showing that more than 70% of the public held negative or stigmatizing attitudes toward patients with mental illnesses (26), especially regarding engaging in closer personal relationships with them (40). One aspect of this attitude is the strong preconception that people with mental illnesses are dangerous, violent, and unpredictable. As a microcosm of social acceptance, nurses shared these discriminatory ideas with the public (41), which were not necessarily connected to their core MHL. This decreases their ability to provide adequate mental health services, which poses a challenge to the successful treatment of patients with mental illnesses.

### 4.2. Differences in MHL level between general and psychiatric nurses

It was shown that psychiatric nurses had higher levels of core MHL, with its elements of knowledge, ability, and recognition than general nurses. A survey of non-mental health departments in four hospitals in China also found that the MHL of non-psychiatric nurses was inadequate, with low identification of common mental disorders [schizophrenia, depression, and generalized anxiety disorder (GAD)] (13). Given that many mental disorders often manifest as somatic discomforts, such as depressive and anxiety disorders, patients may seek help from internal medicine (neurology, gastroenterology, or cardiology) or emergency departments (13, 42). There is also evidence that people experiencing mental disorders are more likely to seek professional help if someone else suggests it (12). In such cases, nurses are the right professionals to facilitate the recognition and help-seeking from patients with mental health problems in general hospitals. Therefore, early identification and timely transfer of patients with mental disorders are crucial to avoid serious consequences in general hospitals. General nurses’ core MHL must be enhanced to strengthen their competence in the prevention and identification of patients with mental health problems.

However, this study showed that nurses in psychiatric hospitals had lower social acceptance of patients with mental illnesses than their colleagues in general hospitals. This result is consistent with previous studies revealing that patients with psychiatric disabilities also reported experiencing negative attitudes from mental health providers (43). This phenomenon might be related to occupational burnout after long-term exposure to work pressure (44) and fear of workplace violence (45), as most psychiatric nurses in this study had longer work durations. It has also been reported that psychiatric nurses experience severe empathic fatigue caused by stressful work environments and professional cognition (46), which may explain the large social distance or low social acceptance of psychiatric nurses among patients with mental health illnesses. As one of the main groups of mental health service providers, psychiatric nurses’ attitudes toward mental illnesses and patients can significantly affect treatment effectiveness and the quality of mental health services. Therefore, the MHLS-SA should be the focus of future educational campaigns. However, more support needs to be provided to psychiatric nurses to improve their acceptance of patients with mental illnesses.

### 4.3. Influential factors of MHL in nurses of general and psychiatric hospitals

This study found other influential factors of MHL besides the hospital type. In line with other studies, all nurses with higher levels of education (e.g., graduate education) showed higher overall and core MHL levels than those with less education (20, 40, 43). The results illustrated that more education increased the opportunities to learn about mental health (28). Therefore, professional education is an effective way to improve nurses’ MHL. This study also found that nurses working in tertiary hospitals had higher levels of MHL, especially core MHL, than those working in secondary hospitals, which may be related to more opportunities for training in the former group.

This study showed that all nurses with higher professional titles had better overall and core MHL, while longer work durations resulted in negative MHL-SA. This indicates that with professional improvement, nurses are more competent in recognizing and dealing with mental problems, which does not mean they would accept being together with patients with mental illness (working or living) because of perceived pressure or empathic fatigue (44–46). Simultaneously, marital status was found to be an independent influential factor for the MHL-SA, with unmarried nurses having a higher acceptance of patients with mental illness, which is consistent with a previous study (40). This phenomenon may be partly due to the emphasis and publicity of mental health in China in recent years, with younger people being easily impacted.

Despite the high rates of mental illness among females, our study did not find sex differences in the level of MHL based on a very small group of male nurses. This result is consistent with that of a previous study on Chinese individuals (47). Some studies have found that females have a higher tendency to seek help, whereas males have significantly more negative mental health attitudes, greater self-stigma about mental illness, and less mental health knowledge than females (26, 39, 43). Therefore, more studies are needed to explore whether sex differences exist in the MHLS-Core and/or MHLS-SA among health professionals.

### 4.4. The relationship between MHL and mental health in nurses of general and psychiatric hospitals

Our results are consistent with those of previous studies, showing that MHL is closely correlated with mental health. Participants experiencing higher levels of depressive or anxiety symptoms had lower overall and core MHL and always preferred non-professionals as their first help-seeking choice (15, 22, 43), or were less likely to recommend help-seeking (38). More specifically, core MHL was positively correlated with the anxiety and depression statuses of non-psychiatric nurses. For psychiatric nurses, total MHL, with its core dimensions and social acceptance, was associated with depressive symptoms, and lower social acceptance was related to anxiety. These facts indicate that it is imperative to improve nurses’ core MHL, including the ability to seek help and recognition in general hospitals; in psychiatric hospitals, it is more important to improve nurses’ social acceptance of mental illnesses as well as core MHL to promote their MHL and mental well-being.

### 4.5. Limitations

First, owing to the cross-sectional design of this study, causality between MHL and mental health or other variables could not be determined. Second, there is a potential for the results to be affected by the abnormal distribution of the dependent variables. Third, this study was conducted in Shanghai, a modern metropolitan area with specific demographic and socioeconomic characteristics. Therefore, not all the results can be generalized to other regions. Furthermore, MHL has many components and is an evolving concept. In addition to the four main domains involved in the current study, positive mental health promotion (6, 7, 19) and first aid skills in a mental health crisis (26) have also been explored and need to be considered in further research.

## 5. Conclusion

This study showed that the overall and core MHL of Chinese nurses were at a moderate level, with social acceptance being relatively low. The core MHL scores of non-psychiatric nurses were lower than those of psychiatric nurses, whereas the social acceptance dimension scores of non-psychiatric nurses were higher than those of psychiatric nurses. There is an urgent need for MHL promotion programs to improve the MHL of clinical nurses. More focus must be given on overall MHL, especially core MHL, for non-psychiatric nurses to enhance their competence in mental health promotion and identification of mental disorders in patients; more emphasis must be placed on the social acceptance of patients with mental illness for psychiatric nurses to improve their provision of professional service. Furthermore, nurses with less education, lower professional titles, longer work durations, working in secondary hospitals, or who are married should be given more attention in promotion programs. Better MHL would be a formula for the improvement of nurses’ own mental health and their competence in providing mental health services.

## 6. Practice and policy implications

This study described the levels and characteristics of MHL in general and psychiatric hospitals, explored the factors influencing MHL, and examined the relationship between MHL and mental health status. The results illustrate that there is room and need for enhancement of the MHL of clinical nurses to handle mental health issues and promote their mental well-being. Mental health education programs or actions emphasizing core MHL, including knowledge, ability, recognition, and social acceptance, need to be implemented to improve nurses’ overall MHL with a greater focus on core MHL for general nurses and social acceptance for psychiatric nurses. Influential factors also help in identifying the important dimensions and targeted nurse groups.

Cultural attitudes toward mental health and illness have served as barriers among the public; therefore, health professionals, especially nurses, should be conscientious in explaining that seeking professional help is not a sign of personal weakness in the presence of mental symptoms or disorders. Improving nurses’ MHL would promote early identification and intervention, as well as improve mental health outcomes at the individual, institutional, and community levels, which may aid mental health practice and support policy development.

## Data availability statement

The raw data supporting the conclusions of this article will be made available by the authors, without undue reservation.

## Ethics statement

The studies involving human participants were reviewed and approved by Institutional Review Board of School of Nursing, Fudan University. The patients/participants provided their written informed consent to participate in this study.

## Author contributions

AW, SJ, and ZS: design, data collection, data analysis, and writing. XS, YZ, and MS: design and data collection. DT and XC: data collection, analysis, and writing. All authors meet the authorship criteria of the latest guidelines of the International Committee of Medical Journal Editors and agree with the manuscript.

## Funding

This research project was funded by the University’s 2021 discipline development funding (FNSYL202107).

## Conflict of interest

The authors declare that the research was conducted in the absence of any commercial or financial relationships that could be construed as a potential conflict of interest.

## Publisher’s note

All claims expressed in this article are solely those of the authors and do not necessarily represent those of their affiliated organizations, or those of the publisher, the editors and the reviewers. Any product that may be evaluated in this article, or claim that may be made by its manufacturer, is not guaranteed or endorsed by the publisher.
